# Can Work Engagement Be a Resource for Reducing Workaholism’s Undesirable Outcomes? A Multiple Mediating Model Including Moderated Mediation Analysis

**DOI:** 10.3390/ijerph16081402

**Published:** 2019-04-18

**Authors:** Liliya Scafuri Kovalchuk, Carmela Buono, Emanuela Ingusci, Francesco Maiorano, Elisa De Carlo, Andreina Madaro, Paola Spagnoli

**Affiliations:** 1 Department of Psychology, University of Campania Luigi Vanvitelli, 8100 Caserta, Italy; liliya.scafurikovalchuk@unicampania.it (L.S.K.); carmela.buono@unicampania.it (C.B.); francesco.maiorano91@gmail.com (F.M.); 2 Department of History, Society and Human Studies, University of Salento, 73100 Lecce, Italy; emanuela.ingusci@unisalento.it (E.I.); elisa.decarlo@unisalento.it (E.D.C.); madaroandreina@gmail.com (A.M.)

**Keywords:** workaholism, work engagement, work–family conflict, emotional exhaustion

## Abstract

This study aimed to explore a possible process explaining the relationship between workaholism and sleep disorders, including two mediators: work–family conflict and emotional exhaustion. Moreover, since a possible buffering role of work engagement was recently proposed against the detrimental effects of workaholism, the aim was to examine the moderating role of work engagement in the relationship between workaholism and several outcomes such as work–family conflict, emotional exhaustion, and sleep disorders. Two models were tested using conditional process analysis for testing direct and indirect effects on a sample of 395 employees: (1) a serial multiple mediation model, and (2) the same serial multiple mediation model including the moderating role of work engagement. Results showed a significant mediating effect of both work–family conflict and emotional exhaustion. Moreover, work engagement moderated the relationship between workaholism and work–family conflict and the relationship between workaholism and emotional exhaustion. This work contributes to the understanding of the process underlying the relationship between workaholism and sleep disorders and to the literature reporting the possible protective role of work engagement on the negative outcomes of workaholism. Practical implications are also discussed.

## 1. Introduction

In recent years, there was increasing attention toward the phenomenon of workaholism, a dysfunctional form of heavy work investment characterized by a set of recurrent behaviors (e.g., working for long hours) and cognitions (e.g., being mentally focused on work activities even when not at work) that have potentially strong negative implications for individual and organizational well-being and vitality [1,2,3]. Sleep disorders constitute one of the health impairment outcomes of being workaholic [4,5,6]. Research on the direct relationship between workaholism and sleep disorders receives consistent attention [5,7,8], whereas studies on the mechanism underlying this relationship are very scant. Accordingly, the first aim of the current study is to contribute to the knowledge of the mechanism underlying the relationship between workaholism and sleep disorders by assuming the mediating role of work–family conflict and emotional exhaustion. 

Another intriguing issue on workaholism is related to its interplay with work engagement, which represents a positive, fulfilling, work-related state of mind where employees bring all their cognitive and emotional energies into work [9,10]. According to the heavy work investment perspective [3], workaholism and work engagement are two faces of the same coin. Thus, in accordance with Loscalzo and Giannini [11] and van Beek and colleagues [12], in the current study, workaholism and work engagement are considered together and crossed conceptually in order to identify different kinds of workaholics. In particular, the second aim of the current study is to examine the interplay of workaholism and work engagement considering two different kinds of workaholics, engaged and disengaged workaholics, in relation to work–family conflict, emotional exhaustion, and sleep disorders.

### 1.1. The Relationship between Workaholism and Sleep Disorders

Sleep is an important healing for recovery from daily strains and, therefore, a prerequisite for optimal daily functioning and health [13]. Walsh and Lindblom [14] suggest that sleep must be sufficiently continuous to be restorative. Sleep problems are a serious threat to the health and well-being of employees, affecting cognitive performance, and mental and physical health [15]. Workaholic employees work hard; thus, they have less opportunity to recover from their work and might have a higher tendency to deplete their resources [16]. Workers showing an inability to stop worrying about work during leisure time and anxiety about work tasks after “office hours” and/or during non-work days show a reduction in sleep quality [7] or difficulty falling asleep [17] or, in general, sleep complaints [5,8,18]. Accordingly, we put forward our first hypothesis.

**Hypothesis** **H1:** *A direct and negative relationship between workaholism and sleep disorders exists.*

### 1.2. The Mediating Role of Work–Family Conflict in the Relationship between Workaholism and Sleep Disorders

Recent technology’s advances contribute to the reduction of the boundaries between work and private life, increasing the likelihood of interference between work and family domains. Clark and colleagues [19] suggest that the utilization of resources in one domain (e.g., work) drains resources available in the other domain; thus, workaholics may experience work–family conflict due to the amount of resources they expend at work, such as cognitive energies. Workaholics experience relatively high work–family conflict [20,21]. Work–family conflict occurs when demands from work and family domains are incompatible, impeding domain performance [22]. Moreover, a number of recent studies found relationships between work–family experiences and sleep quality [23,24,25]. In particular, evidence of the relationship between work–family interference and high levels of daily fatigue and sleep complaints were found [26,27]. Thus, we hypothesize the mediating role of work–family conflict in the relationship between workaholism and sleep disorders.

**Hypothesis** **H2:** *Work-family conflict mediates the relationship between workaholism and sleep disorders.*

### 1.3. The Mediating Role of Emotional Exhaustion in Relationship between Workaholism and Sleep Disorders

Burnout represents one of the negative outcomes of being workaholic [28,29,30,31]. In particular, Clark and colleagues [28] showed that there was a stronger correlation between workaholism and emotional exhaustion and depersonalization. In the current study, we focus on emotional exhaustion, which occurs when the energy, invested excessively in the work, decreases [32]. As burnout, as well as emotional exhaustion, is a stress-related symptom [33,34], associations with sleep quality seem evident. People with high levels of burnout have difficulty falling asleep [35,36]. Giorgi and colleagues [37] showed a circular relationship between burnout and sleep quality, mediated by the effects of personal burnout on impaired sleep quality and of daytime dysfunction on the presence of total burnout. In fact, lower overall burnout levels are associated with better sleep quality [38,39], and better sleep quality predicts lower overall burnout levels [40]. People with high levels of burnout reported all kinds of sleep problems, particularly trouble falling asleep and not refreshing sleep [36,41], as well as sleep of lower quality [42]. Accordingly, we hypothesize the mediating role of emotional exhaustion in the relationship between workaholism and sleep disorders.

**Hypothesis** **H3:** *Burnout mediates the relationship between workaholism and sleep disorders.*

### 1.4. The Mediating Role of Work–Family Conflict and Emotional Exhaustion in the Relationship between Workaholism and Sleep Disorders

As we already posited, investing more time into work has the consequence of reducing resources/energies to be invested at home, and, according to conservation of resources theory (COR) (59), resources are lost [43,44]. According to COR, individuals have a finite amount of resources (time, energy, attention), and, if resources are spent in one role (e.g., work), there is a decrease in the resources available for use in another role (e.g., family). This loss of resources may lead to conflict between work and the family [45]. Several studies showed that the effects of daily stressors from work could have a negative impact on family dynamics [28,46]. Also, the relationship between work–family conflict and burnout was supported by several studies [47]. Actually, employees who experience a conflict between work and family exhaust their energies, increasing the risk of developing burnout. Thus, workaholics tend to use their resources and energies in the workplace, increasing the chances of experiencing work–family conflict. Employees with high levels of workaholism and work–family conflict exhaust their resources and, therefore, experience more stress. If these resources are not recovered, the probability of experimenting burnout increases. Finally, as we previously claimed, the relationship between burnout and sleep disorders was supported [36,40]. According to this literature, we hypothesized a multiple mediation effect of work–family conflict and burnout in the relationship between workaholism and sleep disorders.

**Hypothesis** **H4:** *Work–family conflict and burnout mediate the relationship between workaholism and sleep disorders.*

## 2. The Buffering Role of Work Engagement on Workaholism’s Undesirable Outcomes

Work engagement and workaholism are two work-related states that are indicative of heavy work investment [3,48]. Snir and Harpaz [49] introduced the concept of heavy work investment, which is characterized by two elements: long hours of work and heavy effort. Workaholism and work engagement are subtypes of the heavy work investment: workaholism is based on an addiction to work (an internal, uncontrollable, and stable predictor), while work engagement is an expression of a passion to work (an internal, controllable, and stable predictor). Workaholism is defined as “an irresistible or uncontrollable need to work incessantly” [50]. Schaufeli and colleagues [51] proposed that workaholism is characterized by two elements: working excessively (exceptional amount of time and energy that workaholics devote to the work activity) and working compulsively (a strong and irresistible inner drive to work). Recently, some studies showed a moderate/strong association between these two components [52,53].

However, it was hypothesized that these two components are complementary and not distinct dimensions; therefore, workaholism is often considered as one dimension [54,55]. Workaholics show an exaggerated need to work and it seems impossible for them to repress it, endangering health, reducing their happiness, and deteriorating their interpersonal relationships [51]. On the contrary, work engagement is a positive, fulfilling, work-related state of mind where employees bring all their physical, cognitive, and emotional energies into work [10,56], characterized by vigor (high level of energy and mental resilience while working), dedication (strongly involved in job activities), and absorption (concentrated and happily engrossed in one’s work) [57,58]. A strong correlation was found between the three components [59]; thus, very often, it is proposed as one dimension [60].

Employees characterized by a high level of work engagement work intensively for many hours, as well as workaholics [57], but with passionate involvement [61]. Workaholism and work engagement are associated with distinctive outcomes [62]: workaholism is associated with negative outcomes and work engagement with positive outcomes. Workaholism is associated with high levels of job strain and mental health complaints [12,33,63,64,65,66], job-related negative affects [2,19,46,61,64], poor quality of sleep [5,7,63] and sleeping difficulties [17], more interpersonal conflict at work [67], poorer social relationships [68], burnout [35], and work–family conflict [22,46]. Work engagement is associated with low levels of health complaints [69] and high levels of psychological and physical health [70], high levels of job performance [71], work motivation [12,72], and well-being [73]. Moreover, it is negatively related to burnout [57,74], work–family conflict [75], and sleeping difficulties [76]. Furthermore, the increase in work engagement is included among the measures for health promotion in older workers [77].

Work engagement shows a negative relation with work interference [78,79] in the way that investment in work does not prevent the involvement in various life roles [23,80]. This is in line with Ivy, Siu, and colleagues [81], who showed a positive relationship between work engagement and work family enrichment. Actually, the consequences on the family context of work engagement are in line with the enrichment theory [82], which specifies the conditions under which work and family roles are “allies” rather than “enemies” [83]. In brief, according to the enrichment theory, resources generated in one life role can produce positive consequences in another role [82]. Thus, for example, employees with a high level of work engagement could spill over the positive resources generated in the work context to the family context, and this mechanism could protect them from experiencing work–family conflict. However, before focusing on this specific protective role of work engagement against work–family conflict, we believe it is better to depict the more comprehensive theoretical framework where the possible buffering role of work engagement against the undesirable outcomes of workaholism is inscribed. The first authors who supposed the protective role of work engagement against the negative outcomes of workaholism were van Beek and colleagues [12], who, in accordance with Loscalzo and Giannini [11], found that, in some employees, workaholism and work engagement could both be present. Actually, they described three types of hard workers: workaholic employees, engaged employees, and engaged workaholics. Accordingly, some authors proposed a conceptualization of the interplay between workaholism and work engagement [11,12,84], where work engagement may be a protective factor from the undesirable outcomes of workaholism [64]. These authors inspired their conceptualizations mainly referring to the self-determination theory (SDT) [85]. This theory focuses on the autonomous motivation that is characterized by people being engaged in an activity with a full sense of willingness, will, and choice; furthermore, often, autonomously regulated activities are intrinsically motivated. When the work’s motivation is externally regulated, individuals perceive their behavior as being directly controlled by others, often through contingent rewards and threats; in this case, they talk about “motivation control” that can have negative spillover effects on subsequent performance and work engagement. The SDT shows that workaholism is associated with controlled motivation and work engagement with autonomous motivation [12].

Moreover, van Beek and colleagues [12] showed that engaged workaholics are driven by both controlled and autonomous motivation and that workaholics are driven by controlled motivation. Gillet and colleagues [86] revealed that employees characterized by a high level of autonomous motivation presented high levels of positive affect [84], and that the autonomous motivation buffers the negative effects of controlled motivation [84,87]. Van Beek and colleagues [12] showed that engaged workaholics are associated with less burnout than workaholic employees. The buffering role of work engagement was also examined by the study of Spagnoli and colleagues [6], who found that engaged workaholic women could experience a lower level of job-related negative effects than disengaged workaholic women. However, Gillet and colleagues [86] found contrasting results. Actually, theirs studies reported that the protective role of work engagement on workaholism outcomes was not effective on high levels of workaholism, but a high level of work engagement with a low level of workaholism was associated with positive outcomes.

### 2.1. The Relationship between Workaholism and Work–Family Conflict Moderated by Work Engagement

Despite several studies clearly remarking the positive association between workaholism and work–family conflict [22,46], conflicting evidence was found on the relationship between work engagement and work–family conflict. Some studies showed a negative relationship of work engagement with work interference [78,79], while other studies reported a positive relationship [86]. Regarding the possible consequences of engagement and workaholism, both types of hard workers invest a great deal of their resources into their jobs and their motivation is so different that the long-term consequences of their high commitment to work are also different for their work-to-family balance [59]. Hakanen and colleagues [59] showed that work engagement is associated with work-to-family enrichment and also predicted less work–family conflict in the long term. Gillet and colleagues [86] showed that work engagement is associated with a decrease in work–family conflict; however, it did not protect employees against the negative effects of workaholism on work–family conflict when the levels of workaholism are high. Theoretically, engagement does not exist as an isolated resource but is often accompanied by, for example, positive affect, stronger self-efficacy, and perceiving the positive results of one’s accomplishments; thus, it is likely that these positive experiences will also spill over into family life. Therefore, in the present study, we hypothesize a moderated effect by work engagement in the relationship between workaholism and work–family conflict. More particularly, we envisage that high levels of work engagement, with both high and low levels of workaholism, may reduce the levels of work–family conflict.

**Hypothesis** **H5:** *Work engagement moderates the relationship between workaholism and work–family conflict, in the way that high levels of work engagement significantly reduce the level of work–family conflict when workaholism is high.*

### 2.2. The Relationship between Workaholism and Burnout Moderated by Work Engagement

A positive association between workaholism and burnout was clearly demonstrated [30,87]. Work engagement is negatively related to burnout [74,86]. Gillet and colleagues [86] showed that work engagement was associated with a decrease in burnout, but high levels of work engagement associated with a high level of workaholism are related with a high level of burnout. Therefore, evidence of the buffering role of work engagement was not found. However, taking into consideration the study of van Beek and colleagues [12], engaged workaholics showed a lower level of burnout than workaholic employees and they had a higher level of burnout than engaged employees. Thus, according to van Beek and colleagues [12], in the present study, we hypothesize the moderated effect of work engagement in the relationship between workaholism and burnout. Specifically, we envisage that high levels of work engagement, with both high and low levels of workaholism, may reduce the levels of burnout.

**Hypothesis** **H6:** *Work engagement moderates the relationship between workaholism and burnout, in the way that high levels of work engagement significantly reduce the level of burnout when workaholism is high.*

### 2.3. The Relationship between Workaholism and Sleep Disorders Moderated by Work Engagement

Workaholism had a positive association with a poor quality of sleep and sleep disorders (insufficiency of sleep, excessive daytime sleepiness at work, difficulty awakening in the morning, and tiredness upon awakening) [1,5,76]. Workaholics, due to excessive and compulsive working, are not likely to be able to detach mentally from work and, therefore, may be prone to sleep problems and continue working even when they feel sick [30]. Moreover, the study of Spagnoli and colleagues [6] showed that workaholics, given that they experience a high level of negative affect and anxiety, have sleep disorders. Work engagement is positively linked to recovery experience and the engaged employees sleep well most of the time [88]. Moreover, engaged workers appear to be able to recovery process after working [89]. Thus, in the present study, we hypothesize a moderating role of work engagement in the relationship between workaholism and sleep disorders. Specifically, high levels of work engagement, with both high and low levels of workaholism, may reduce sleep disorders.

**Hypothesis** **H7:** *Work engagement moderates the relationship between workaholism and sleep disorders, in the way that high levels of work engagement significantly reduce the level of sleep disorders when workaholism is high.*

## 3. Materials and Methods

### 3.1. Recruitment

The sample was selected based on the profession that participants carried out, focusing on jobs with a high risk of workaholism [90].

A total of 395 Italian participants took part in the study. The sample consisted of 245 (62% women) and 150 (38% men). Their age ranged from 18 to 64 years (M = 39.98; SD = 12.107). The profession categories are distributed as follows: teachers, 33.3%; freelancers, 35.5%; educators, 4.4%; clerks 14.7%; managers, 5.1%; doctors, 2.5%; researchers, 4%. They were employed in both the private sector (54.4%) and public sector (42.4%). Educational level was distributed as follows: 3.2% middle school; 36% high school; and 57.6% bachelor’s degree or higher. Tenure ranged from a few months to 42 years (mean = 12.41; SD = 11.25).

### 3.2. Questionnaire Administration

A positive association between workaholism and burnout was clearly demonstrated [30,87].

Graduated students on work and organizational psychology courses took part in the data collecting phase of the study as part of their master’s degree thesis assignment. Firstly, they were asked to identify in their social network a limited number of employees to be involved in the study. Then, an email including the link to the online questionnaire was sent to them so that they could forward it to the identified employees.

### 3.3. Measures

#### 3.3.1. Workaholism

Workaholism was measured using the 10-item version of the Dutch Work Addiction Scale (DUWAS) previously adapted and validated in Italy [91]. The DUWAS investigates the respondent’s feelings about his/her work, which reflect the two components of workaholism (i.e., working compulsively (WC) and working excessively (WE)). Example items are the following: “*I feel that there is something inside me that drives me to work hard*” (WC) and “*I stay busy and keep many irons in the fire*” (WE). Responses are given on a five-point scale varying from 1 (“never or almost never”) to 5 (“almost always or always”). Since the two workaholism components were strongly correlated (*r* = 0.67, *p* < 0.001), we derived an overall workaholism score following the examples of other scholars [12,91].

#### 3.3.2. Work Engagement

Work engagement was measured with the nine-item Utrecht Work Engagement Scale adapted in Italy by Balducci and colleagues [92]. Participants were asked to respond on a five-point scale ranging from “never” to “every day” with regard to how frequently they experienced the feeling.

#### 3.3.3. Work–Family Conflict

Work–family conflict was assessed with the five-item work–family conflict (WFC) scale previously adapted in Italy by Colombo and Ghislieri [93]. The WFC explores the inter-role conflict in which the job demands and job strain interfere with performing family-related responsibilities. Participants were asked to respond on a five-point scale ranging from “agree” to “disagree”. An item example is as follows: “*The demands of my work interfere with my home and family life*”.

#### 3.3.4. Emotional Exhaustion

Six items from Maslach Burnout Inventory, previously adapted in Italy, were used to measure emotional exhaustion [94]. Respondents were asked to rate the frequency of effects on a five-point Likert scale from 1 (never) to 5 (every day). An item example is as follows: “*I feel emotionally drained from my work*”.

#### 3.3.5. Sleep Disorders

The dimension of sleep disorders was assessed using five items from the Mini Sleep Questionnaire (MSQ), adapted and validated in Italy on the general population by Natale et al. [95]. Respondents used a five-point Likert scale ranged from 1 “never” to 5 “always” to evaluate their sleep quality. Item examples are as follows: “difficulty falling asleep”; “waking up too early”.

#### 3.3.6. Workload

Workload was measured using four items (e.g., “*I have to work very fast*”) from the Job Content Questionnaire [96]. Responses were given on a five-point scale varying from 1 “strongly disagree” to 5 “strongly agree”.

#### 3.3.7. Perfectionism

The trait of perfectionism was measured using the eight-item version of the Revised Almost Perfect Scale (SAPS) developed by Rice et al. [97]. The SAPS contains two subscales which investigate two essential elements of perfectionism: standards (i.e., “*I set very high standards for myself*”) and discrepancy (i.e., “*Doing my best never seems to be enough*”). Participants replied using a five-point scale ranging from 1 “strongly disagree” to 5 “strongly agree”.

Since only the workload and perfectionism scales are yet to be adapted in Italy, a rigorous translation process was conducted following Brislin’s procedure of back-translation [98]. For each variable in the study, Cronbach’s alpha coefficients are reported (see Table 1).

### 3.4. Ethical Aspects

The procedure was in accordance with the standards of the national law of data treatment followed by the University of Campania (Italy). Since there was no medical treatment or other procedures that could cause psychological or social discomfort to participants, who were all adult healthy subjects anonymously involved, additional ethical approval was not required according to the institution. The research was conducted in line with the Helsinki Declaration, as well as the data protection regulation of Italy (Legislative Decree No. 196/2003). Participation in the research was voluntary and not rewarded; data collection and analysis were anonymous. A cover letter attached to the questionnaire provided information about the study aims, guarantees about anonymity, voluntary participation, and data treatment, and instructions for filling out the questionnaire. By agreeing to fill out the questionnaire, all study participants provided their informed consent.

### 3.5. Data Analysis

Cronbach’s alpha coefficient and zero-order correlations were used to assess the internal consistencies of the scale and to examine the associations between variables. The hypotheses concerning direct, mediated, and moderated effects were tested through conditional process analysis based on ordinary least squares (OLS) regression using a bootstrapping technique [99], a nonparametric resampling procedure that does not assume normality and involves the extraction of several thousand sub-samples (5000 in our case) from the dataset. Through bootstrapping, the distribution of effects is empirically approximated and used for calculating confidence intervals [100].

Specifically, the models examined in the current study correspond to the conceptual model numbers 6 and 85 of Hayes templates [99]. Gender, age, tenure, workload, and perfectionism were inserted in the model as control variables.

## 4. Results

**Figure 1:**
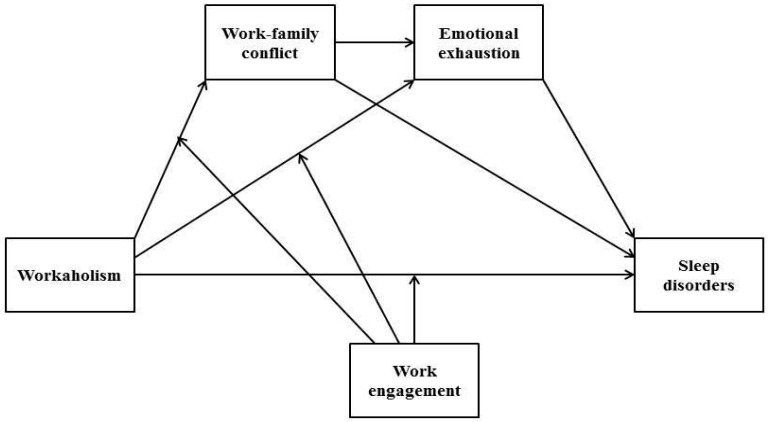
Mediation effect of work–family conflict and emotional exhaustion in the relationship between workaholism and sleep disorders, including interactions between workaholism and work engagement on work–family conflict, emotional exhaustion, and sleep disorders.

Table 1 shows the zero-order correlations among study variables and their reliability measured by Cronbach’s alpha coefficient. Workaholism was positively and statistically correlated to the two subscales of perfectionism, workload, work engagement, work family conflict, emotional exhaustion, and sleep disorders, whereas it was negatively and significantly correlated to age. Strong positive correlations were found between work engagement, the standard subscale of perfectionism, and workload. Moreover, work engagement was positively and significantly correlated to gender, whereas it was negatively correlated to the discrepancy subscale of perfectionism, work–family conflict, and emotional exhaustion. The reliability coefficients expressed by the Cronbach α ranged from 0.67 (workload) to 0.90 (work engagement), indicating satisfactory internal reliability for all variables except for workload (see Table 1). However, Cronbach’s alpha coefficient can be smaller if the number of items in the scale is fewer than 10 [101]. Thus, because workload is composed of four items, this measure was considered in the analysis. Table 2 reports the results for the conditional process analysis conducted on the two models: Model 1, which represents a serial multi-mediation model where work–family conflict and emotional exhaustion mediate the relationship between workaholism and sleep disorders; and Model 2, which represents Model 1 with the addition of moderation by work engagement in the three investigated directions (see Figure 1).

In Model 1 we estimated all the path coefficients, simultaneously controlling for gender, age, tenure, workload, and perfectionism. In this model, we tested for a three-path mediated effect [99], where the first path concerned the relationship between workaholism and sleep mediated by work–family conflict; the second path concerned the relationship between workaholism and sleep mediated by emotional exhaustion; finally, the third path concerned both these mediators in the previously named relationship. A significant direct effect of workaholism on sleep disorders was found (β = 0.10, LLCI = 0.00, ULCI = 0.20). Thus, hypothesis H1 was supported. Moreover, the results showed the existence of a significant partial mediating effect of work–family conflict and emotional exhaustion in the relationship between workaholism and sleep disorders (indirect effect = 0.04, LLCI = 0.02, ULCI = 0.07). In particular, the mediating effect explained 28% of the variance of the direct effect (Model 1: *R*^2^ = 0.28). Furthermore, only one of the control variables (age) had a significant, albeit weak effect in the tested model (β = 0.01, LLCI = 0.00, ULCI = 0.19). Thus, hypothesis H4 was supported. No support was found for hypothesis H2, although the result was close to significant (indirect effect = 0.04, LLCI = −0.01, ULCI = 0.08). Also, hypothesis H3 of the mediating role of emotional exhaustion in the relationship between workaholism and sleep disorders was not supported by the evidence (indirect effect = 0.01, LLCI = −0.01, ULCI = 0.05). Subsequently, in Model 2, a moderated mediation model was tested to examine the moderating effects of work engagement. The mediating effect in Model 2 explained 55% of the variance (Model 2: *R*^2^ = 0.55). In this model, the indirect effect of both mediators work–family conflict and emotional exhaustion on sleep was moderated by work engagement (index of moderated mediation = −0.02, BootLLCI = −0.04, BootULCI = −0.01). This path was significant at low (β = 0.05, BootLLCI= 0.02, BootULCI = 0.08); medium (β = 0.03, BootLLCI = 0.01, BootULCI = 0.06) and high (β = 0.02, BootLLCI= 0.01, BootULCI = 0.04) level of moderator.

In Model 2, evidence of a significant interaction between workaholism and work engagement on work–family conflict was found (β = −0.20, LLCI = −0.35, ULCI = −0.06), as well as a moderating effect of work engagement in the interaction between workaholism and emotional exhaustion (β = −0.21, LLCI = −0.33 ULCI = −0.09). Some of the control variables such as gender (β = 0.17, LLCI = 0.05, ULCI = 0.30), tenure (β = 0.01 LLCI = 0.00, ULCI = 0.02), workload (β = 0.25, LLCI = 0.14, ULCI = 0.36), and the discrepancy subscale of perfectionism (β = 0.017, LLCI = 0.08, ULCI = 0.25) had a significant effect in the tested model.

**Figure 2:**
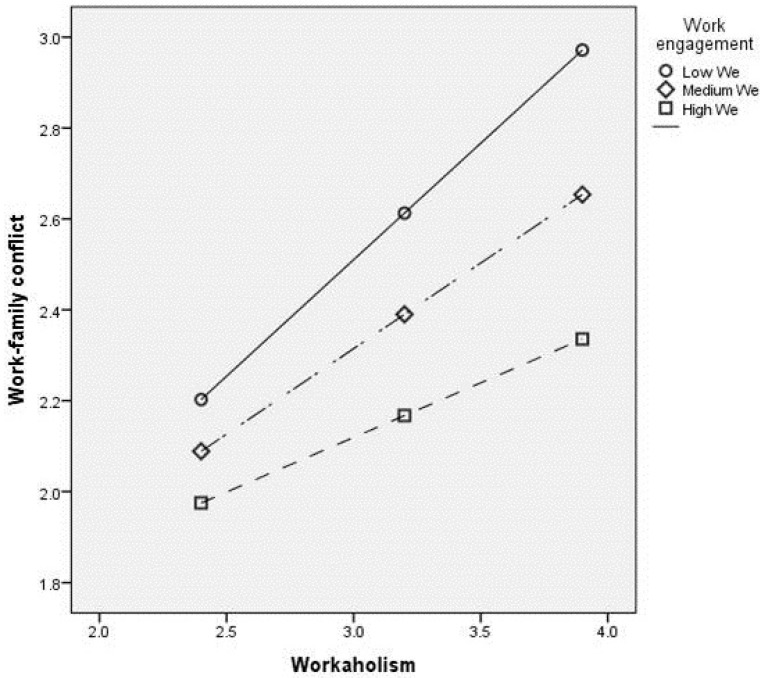
Plot of the interaction between workaholism and work engagement on work–family conflict.

Figure 2 reports the plots regarding the interaction between workaholism and work engagement on work–family conflict. Following Hayes [99], the values of workaholism were observed at the 16th, 50th, and 84th percentiles in work engagement. In particular, when work engagement is low and workaholism is high, work–family conflict is significantly higher than when work engagement is high. Moreover, a simple slope analysis revealed that, although the effect of workaholism on work–family conflict was significant at low (β = 0.51 *p* < 0.001), medium (β = 0.38 *p* < 0.001), and high (β = 0.24, *p* < 0.05) levels of the moderator, the effect of the predictor was significantly higher when work engagement was low.

**Figure 3:**
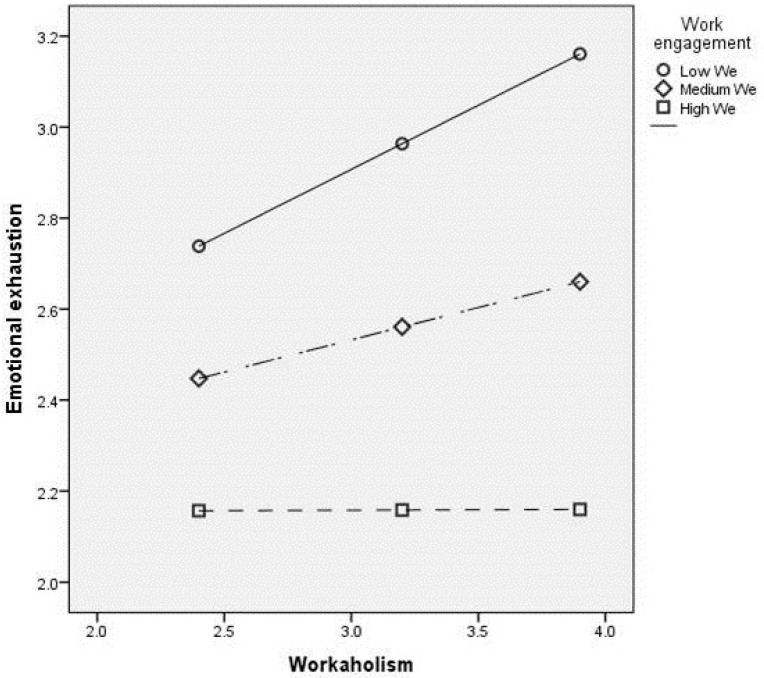
Plot of the interaction between workaholism and emotional exhaustion, moderated by work engagement.

Additionally, as hypothesized, work engagement moderated the relationship between workaholism and emotional exhaustion. In particular, when work engagement is low and workaholism is high, the level of emotional exhaustion is significantly higher than when work engagement is high. In fact, a simple slope analysis (Figure 3), revealed that, although the effect of workaholism on emotional exhaustion was positive and significant at low (β = 0.28, *p* < 0.001) and medium (β = 0.14, *p* < 0.05) levels of the moderator, the effect of the predictor was significantly higher when work engagement was low, and it was non-significant when work engagement was high (β = 0.00, *p* = 0.98). This suggests that work engagement acts as a protecting factor among workaholics against emotional exhaustion. Thus, hypothesis H6 was supported.

Finally, the hypothesis of the moderating role of work engagement between workaholism and sleep disorders (hypothesis H7) was not supported by the evidence (β = 0.01, LLCI = 0.12, ULCI = 0.15).

## 5. Discussion

The aim of the current study was twofold: (1) examining the mediating role played by both work–family conflict and emotional exhaustion in the relationship between workaholism and sleep disorders; (2) investigating the moderating role of work engagement in the relationship between workaholism and three outcomes, such as work–family conflict, emotional exhaustion, and sleep disorders. Regarding the first aim, results supported the multiple mediating model. This contributes to the literature on the relationship between workaholism and sleep disorders by highlighting a possible process through which workaholics experience sleep disorders. In particular, since workaholics work excessively at the expense of private life, according to the literature [21,28,30,102], they experience a high level of work–family conflict, which, in turn, might lead to a high level of emotional exhaustion and, thus, flowing to sleep disorders [35,36,37]. Considering the second aim of the current study, evidence of the buffering role of work engagement was found against work–family conflict and emotional exhaustion. This result is in line with van Beek and colleagues [12], who found a protective role of work engagement against burnout, in the way that engaged workaholics experienced less burnout than disengaged workaholics. However, this is in contrast to the study of Gillet and colleagues [86], who reported that when a high level of workaholism is present, work engagement has no effect on the undesirable outcomes of workaholism, such as work–family conflict and burnout. Rather, the results of Gillet and colleagues [86] reported that high levels of work engagement and workaholism were related to worse outcomes. According to Gillet and colleagues [86], our results reported that work engagement did not play a buffering role against sleep disorders. A separate discussion deserves the issue concerning work–family conflict. Since both workaholics and engaged employees work excessively, Loscalzo and Giannini [11] assumed that engaged workaholics should not differ from disengaged workaholics in the way they experience work–family conflict. In line with this assumption, Gillet and colleagues [86] found that high levels of work engagement and workaholism were related to a higher level of work–family conflict. On the contrary, in the current study, evidence of a protective role of work engagement was found against work–family conflict. These results support our hypothesis regarding a possible spillover effect, which would allow a crossover of positive resources between the work and the family domains. In other words, according to the enrichment theory [103], if, for example, one is happy and satisfied regarding his/her own work, although he/she works excessively, he/she will easily recover from working by spilling the positive emotions over from the work to family domain.

Finally, several relevant variables were included in the study as control variables. As it can be noted, perfectionism, workload, and gender had a significant effect in some steps of the analyses. Interestingly, despite these significant effects, most of the hypothesized relationships in the tested models were supported, strengthening the obtained results. Particularly, perfectionism’s relationship with both workaholism and work engagement is intriguing. Actually, while the standard sub-dimension was positively related to both workaholism and work engagement, the discrepancy sub-dimension was positively related to workaholism and negatively related to work engagement. Thus, it seems that the critical perfectionism sub-dimension is discrepancy, since it could significantly contribute to the development of the negative form of heavy work investment. In sum, although some insightful evidence was found, the avenue of understanding the interplay between workaholism and work engagement remains to be fully elucidated.

### 5.1. Limitations and Future Directions

Despite our study shedding some light on a possible mechanism through which workaholism is related to sleep disorders, and despite it contributing to the literature on the possible buffering role of work engagement on the undesirable outcomes of workaholism, some important limitations need to be taken into account. Firstly, the cross-sectional nature of the study does not allow causal inference between the variables. Thus, although the multiple mediating models at the core of the study stem from sound theoretical basis, the results should be interpreted with caution. Further studies should confirm the mediating model longitudinally. Secondly, since self-report measures were adopted, the results might be influenced by the participants’ acquiescence and need for social desirability, and the emerged parameter estimates may have been contaminated by common method bias [101]. Future studies should adopt multisource and objective measures. For example, as far as sleep disorders are concerned, actigraphy could be used for detecting more objective measures of quality and quantity of sleep. Thirdly, as it regards work–family conflict, data from other members of the family could be collected. Fourthly, future research could also focus on the generational differences and on the role of job crafting as a positive moderator to reduce the undesirable outcomes of workaholism. For example, age-oriented human resource (HR) practices and, in general, the diversity management of HR, which are linked to job crafting behaviors, could lead to a positive organizational diversity climate [104,105]. Since job crafting behaviors seem to be strongly related to work engagement [106,107], they can be considered a useful strategy to reduce undesirable outcomes of workaholism and to improve positive organizational outcomes. Finally, the snowball technique for data collection contravenes many of the assumptions supporting conventional notions of random selection and representativeness. As we did not randomly assign the participants, potential biases should be kept in mind when interpreting our results. However, it is not uncommon to use such a sampling strategy in organizational research [108].

### 5.2. Practical Implications

The present study extends and enhances the current knowledge on the phenomenon of work engagement and its interaction with workaholism on some important negative outcomes such as work–family conflict and emotional exhaustion. This study can be potentially valuable to human resource (HR) managers and career counsellors, as it highlights the protecting role of work engagement against negative consequences of workaholism. In particular, even when workaholism is high among employees, and it is known that workaholics devote a lot of energy and time to their work and they neglect their personal affairs and family duties that in turn leads them to work–family conflict, high levels of work engagement might significantly reduce this negative outcome. Furthermore, our results suggest that work engagement could play an important role in workaholics to reduce burnout, particularly the dimension of emotional exhaustion. Thus, taking together this evidence, several recommendations can emerge. Firstly, organizations should give priority to preventing workaholism and its negative consequences by promoting suitable organizational conditions and autonomous motivation at work, which would lead to an increase in work engagement [12]. Since work engagement is linked to both individual and organizational benefits, it should be considered as a precious resource for promoting well-being at all levels. Moreover, organizations could encourage an organizational culture that strongly dissuades excessive working and ensures that the number of tasks for which employees are responsible are reasonable and compatible with their family duties. The implementation of practices that reduce work–family conflict, for example, with flexible-time work policies, is linked to higher work commitment and lower organizational health-related costs of current employees [109].

## 6. Conclusions

The current study provided evidence of a possible process through which workaholism is related to sleep disorders and of the buffering role of work engagement against undesirable outcomes of workaholism such as work–family conflict and emotional exhaustion. Although they are encouraging and interesting results, they should be supported using a longitudinal approach. Fostering work engagement might be a useful and effective intervention for buffering the negative consequences of workaholism in terms of work–family conflict and emotional exhaustion.

## Figures and Tables

**Table 1:** Descriptions, inter-correlations, and reliabilities of the study variables.

Variables	Mean	SD	1	2	3	4	5	6	7	8	9	10	11
1. Gender ^#^	1.62	0.49											
2. Age	39.98	12.11	−0.03										
3. Tenure	12.41	11.25	−0.01	0.80 **									
4. Perfectionism standard	3.63	0.80	−0.03	−0.26 **	−0.22 **	**(0.86)**							
5. Perfectionism discrepancy	2.68	0.90	−0.01	−0.22 **	−0.18 **	0.28 **	**(0.84)**						
6. Workload	3.76	0.66	−0.10	−0.04	−0.04	0.30 **	0.03	**(0.67)**					
7. Workaholism	3.18	0.73	0.01	−0.12 *	−0.05	0.27 **	0.35 **	0.40 **	**(0.83)**				
8. Work engagement	3.85	0.69	0.11 *	0.06	0.07	0.31 **	−0.26 **	0.29 **	0.14 **	**(0.90)**			
9. Work–family conflict	2.38	0.82	−0.04	−0.06	−0.02	0.12 *	0.27 **	0.18 **	0.40 **	−0.16 **	**(0.88)**		
10. Emotional exhaustion	2.58	0.88	0.00	0.01	0.06	0.06	0.41 **	0.19 **	0.34 **	−0.43 **	0.53 **	**(0.89)**	
11. Sleep disorders	2.35	0.76	0.03	0.12 *	0.12 *	0.03	0.25 **	0.17 **	0.34 **	−0.08	0.35 **	0.45 **	**(0.82)**

# Gender was coded as 1 = men and 2 = women; ** *p* < 0.001; * *p* < 0.05; Cronbach’s alphas are in the diagonal in bold.

**Table 2:** Results of conditional process analysis.

Models	β	LLCI ^⌘^	ULCI	*R* ^2^
**a. Model 1: Mediation of work–family conflict and emotional exhaustion in the relationship between workaholism and sleep disorders**				0.18 **
Outcome variable: Work–family conflict				
Workaholism	0.36 **	0.24	0.48	
Covariate: Gender	−0.07	−0.23	0.08	
Covariate: Age	−0.00	−0.01	0.01	
Covariate: Tenure	0.00	−0.01	0.01	
Covariate: Workload	0.07	−0.06	0.19	
Covariate: Perfectionism standard	−0.03	−0.13	0.08	
Covariate: Perfectionism discrepancy	0.14 *	0.05	0.24	
**b. Model 1: Mediation of work–family conflict and emotional exhaustion in the relationship between workaholism and sleep disorders**				0.41 **
Outcome variable: Emotional exhaustion				
Workaholism	0.06	−0.06	0.17	
Work–family conflict	0.47 **	0.37	0.56	
Covariate: Gender	0.07	−0.07	0.21	
Covariate: Age	−0.00	−0.01	0.01	
Covariate: Tenure	0.01	−0.00	0.02	
Covariate: Workload	0.16 *	0.04	0.27	
Covariate: Perfectionism standard	−0.12 *	−0.21	−0.02	
Covariate: Perfectionism discrepancy	0.32 **	0.23	0.40	
**c. Model 1: Mediation model of work–family conflict and emotional exhaustion in the relationship between workaholism and sleep disorders**				0.28 **
Outcome variable: Sleep disorders				
Workaholism	0.18 *	0.07	0.29	
Work–family conflict	0.10	−0.00	0.20	
Emotional exhaustion	0.25 **	0.15	0.34	
Covariate: Gender	0.06	−0.08	0.20	
Covariate: Age	0.01 *	0.00	0.19	
Covariate: Tenure	−0.00	−0.01	0.01	
Covariate: Workload	0.07	−0.04	0.18	
Covariate: Perfectionism standard	−0.04	−0.14	0.05	
Covariate: Perfectionism discrepancy	0.08	−0.01	0.16	
**Indirect effect through work–family conflict**	0.04	−0.01	0.08	
**Indirect effect through work emotional exhaustion**	0.01	−0.01	0.05	
**Indirect effect through work–family conflict and emotional exhaustion**	0.04 *	0.02	0.07	
**d. Model 2: Mediation model including interaction between workaholism and work engagement on work–family conflict**				0.25 **
Outcome variable: Work–family conflict				
Workaholism	1.17 **	0.62	1.72	
Work engagement	0.32	−0.13	0.77	
Workaholism × work engagement	−0.20 *	−0.35	−0.06	
Covariate: Gender	−0.01	−0.16	0.14	
Covariate: Age	0.00	−0.01	0.01	
Covariate: Tenure	0.00	−0.01	0.01	
Covariate: Workload	0.11	−0.02	0.23	
Covariate: Perfectionism standard	0.08	−0.03	0.19	
Covariate: Perfectionism discrepancy	0.06	−0.04	0.15	
**e. Model 2: Mediation model including interaction between workaholism and work engagement on emotional exhaustion**				0.55 **
Outcome variable: Emotional exhaustion				
Workaholism	0.96 **	0.49	1.43	
Work–family conflict	0.34 **	0.26	0.43	
Work engagement	0.07	−0.31	0.45	
Workaholism × work engagement	−0.21 **	−0.33	−0.09	
Covariate: Gender	0.17 *	0.05	0.30	
Covariate: Age	0.00	−0.01	0.01	
Covariate: Tenure	0.01 *	0.00	0.02	
Covariate: Workload	0.25 **	0.14	0.36	
Covariate: Perfectionism standard	0.08	−0.01	0.17	
Covariate: Perfectionism discrepancy	0.17 **	0.08	0.25	
**f. Model 2: Model including interaction between workaholism and work engagement on sleep disorders**				0.28 **
Outcome variable: Sleep disorders				
Workaholism	0.09	−0.43	0.62	
Work–family conflict	0.10 *	0.00	0.20	
Emotional exhaustion	0.29 **	0.18	0.40	
Work engagement	0.07	−0.34	0.48	
Workaholism × work engagement	0.02	−0.12	0.15	
Covariate: Gender	0.04	−0.10	0.17	
Covariate: Age	0.01	−0.00	0.02	
Covariate: Tenure	−0.00	−0.01	0.01	
Covariate: Workload	0.04	−0.08	0.16	
Covariate: Perfectionism standard	−0.08	−0.18	0.02	
Covariate: Perfectionism discrepancy	0.09 *	0.00	0.18	
**Index of moderated mediation:**	**Index**			
Index of conditional moderated mediation by work engagement: Mediation model including interaction between workaholism and work–family conflict on sleep	−0.02	−0.05	0.00	
Index of conditional moderated mediation by work engagement: Mediation model including interaction between workaholism and emotional exhaustion on sleep	−0.06 *	−0.11	−0.02	
Index of conditional moderated mediation by work engagement: Mediation model of work–family conflict and emotional exhaustion in the relationship between workaholism and sleep	−0.02 *	−0.04	−0.01	

* *p* < 0 05; ** *p* < 0.001; ^⌘^ LLCI, ULCI: lower and upper levels for confidence interval.
